# Sensor Sensibility—HIV-1 and the Innate Immune Response

**DOI:** 10.3390/cells9010254

**Published:** 2020-01-20

**Authors:** Xin Yin, Simon Langer, Zeli Zhang, Kristina M. Herbert, Sunnie Yoh, Renate König, Sumit K. Chanda

**Affiliations:** 1 Immunity and Pathogenesis Program, Infectious and Inflammatory Disease Center, Sanford Burnham Prebys Medical Discovery Institute, 10901 North Torrey Pines Road, La Jolla, CA 92037, USA; xyin@sbpdiscovery.org (X.Y.); simon.langer@boehringer-ingelheim.com (S.L.); zzhang@lji.com (Z.Z.); kherbert@sbpdiscovery.org (K.M.H.); syoh@sbpdiscovery.org (S.Y.); Renate.Koenig@pei.de (R.K.); 2 Boehringer Ingelheim Pharma GmbH & Co. KG, 55216 Ingelheim am Rhein, Germany; 3 Division of Vaccine Discovery, La Jolla Institute for Immunology, 9420 Athena Cir, La Jolla, CA 92037, USA; 4 Host-Pathogen Interactions, Paul-Ehrlich-Institut, 63225 Langen, Germany

**Keywords:** HIV-1, immune cells, innate immunity, sensing, evasion strategies, PAMP, PRR

## Abstract

Innate immunity represents the human immune system’s first line of defense against a pathogenic intruder and is initiated by the recognition of conserved molecular structures known as pathogen-associated molecular patterns (PAMPs) by specialized cellular sensors, called pattern recognition receptors (PRRs). Human immunodeficiency virus type 1 (HIV-1) is a unique human RNA virus that causes acquired immunodeficiency syndrome (AIDS) in infected individuals. During the replication cycle, HIV-1 undergoes reverse transcription of its RNA genome and integrates the resulting DNA into the human genome. Subsequently, transcription of the integrated provirus results in production of new virions and spreading infection of the virus. Throughout the viral replication cycle, numerous nucleic acid derived PAMPs can be recognized by a diverse set of innate immune sensors in infected cells. However, HIV-1 has evolved efficient strategies to evade or counteract this immune surveillance and the downstream responses. Understanding the molecular underpinnings of the concerted actions of the innate immune system, as well as the corresponding viral evasion mechanisms during infection, is critical to understanding HIV-1 transmission and pathogenesis, and may provide important guidance for the design of appropriate adjuvant and vaccine strategies. Here, we summarize current knowledge of the molecular basis for sensing HIV-1 in human cells, including CD4^+^ T cells, dendritic cells, and macrophages. Furthermore, we discuss the underlying mechanisms by which innate sensing is regulated, and describe the strategies developed by HIV-1 to evade sensing and immune responses.

## 1. Introduction

Pattern recognition receptors (PRRs) act as microbial sensors for the host to initiate an immediate innate immune response through binding to invariant features of microbes, termed pathogen associated molecular patterns (PAMPs) [1,2,3]. Once a PAMP is recognized by a PRR, a cascade of innate signaling pathways is activated, leading to induction of the host anti-viral response. This includeds the secretion of interferons (IFNs), which is a potent inhibitor of a broad spectrum of viral pathogens [4,5,6]. IFNs then functions both in an autocrine and paracrine manner to drive highly coordinated expression of interferon stimulated genes (ISGs) with antiviral properties [4]. Relevant to HIV-1 infection, ISGs such as the restriction factors APOBEC3, SAMHD1, TRIM5α, Tetherin, and SERINC5 are induced to restrict viral replication at multiple stages in the replication cycle [7]. In addition to providing a first line of defence against pathogen infection, innate immune responses are pivotal in priming the adaptive immune responses through the induction of antigen specific T cell activation and promotion of humoral immunity. Thus, understanding mechanisms by which the innate immune system recognizes and responds to HIV will be instructive in the design of HIV-1 vaccines and/or adjuvants [8,9,10].

Activation of innate immune responses has been observed in individuals during acute HIV infection, as well as macaques infected with simian immunodeficiency virus (SIV) [11,12,13]. In individuals infected with HIV-1, systemic virus dissemination is often accompanied by a transient type I IFN spike early during infection [12]. Importantly, studies with HIV-1 variants from the site of transmission known as transmitted/founder (T/F) viruses revealed T/F viruses have been reported to be, on average less, sensitive to IFNs than chronic systemic viruses, suggesting a selective impact of IFN controlling virus transmission [11,12]. Multiple studies have also demonstrated that HIV-1 infection induces innate immune responses in vitro/ex vivo, particularly in monocyte derived dendritic cells (MDDCs) and macrophages [14,15]. However, detection of innate immune responses to HIV-1, even in these cell types, requires specialized experimental conditions. For example, at low multiplicity of infection (MOI), co-infection/expression of SIV virus like particles (VLPs) bearing the accessory protein Vpx, is required for detection of an innate response to HIV-1 [16,17]. Vpx alleviates the block of early reverse transcription in highly restrictive cells such as DCs, which likely increases the cytosolic pool of HIV-1 cDNA, thereby enabling a detectable innate responses [18,19]. Alternatively, HIV-1 infection at high MOIs can be sufficient for a detectable innate response, independently of Vpx [14,15,16,20]. In addition, it has been observed that HIV-1 harboring capsid mutations can trigger a more robust innate immune response in comparison to wild type virus [14,21]. The potential basis of this enhanced immunogenicity will be discussed in subsequent sections. Collectively, this evidence suggests that innate sensing of HIV-1 infection is likely to be a rare event, but still can have a profound impact on viral transmission and systemic pathology.

Exploration of the molecular basis of HIV-1 innate immune sensing has been an ongoing topic of intense interest. To date, multiple families of PRRs have been implicated as sensors responsible for detecting HIV-1 infection, which include toll-like receptors (TLRs), RIG-I-like receptors (RLRs), and cytosolic DNA sensors (Table 1) [16,22,23,24]. The mechanistic details for initiation and regulation of HIV-1 innate sensing have been extensively studied, but remain controversial regarding the PAMP, innate sensors, and regulators involved in physiologically-relevant sensing processes [9,10,25]. Moreover, HIV-1 has evolved elaborate strategies to circumvent immune surveillance and response. A growing body of evidence has revealed that HIV-1 infection manipulates antiviral immune responses through shielding encoded PAMPs, disabling innate immune regulators, modulating the transcription of innate immune effector genes, and/or antagonizing the antiviral effect of ISGs [26,27,28,29]. Here, we seek to highlight the recent findings regarding the innate sensing of HIV-1 infection and discuss the mechanisms by which the innate immune system have been observed to be engaged and regulated by HIV-1. In addition, we summarize strategies exploited by HIV-1 to evade sensing by the innate immune response. A thorough understanding of the interplay between host innate immunity and HIV-1 infection will reveal mechanistic insights into HIV-1 transmission and pathogenesis, and can inform novel strategies for preventing HIV-1 infection [30,31].

## 2. Innate Immune Sensors of HIV-1 Infection

### 2.1. HIV-1 RNA Sensors

Upon HIV-1 infection, two strands of genomic single-stranded (ss) RNA are encapsulated in virions. These nucleic acids, as well as HIV-1 transcripts that are generated from proviruses, potentially act as PAMPs [23,24,32,33,34,35,36,37]. Both RLR family members, including retinoic acid inducible gene I (RIG-I) and melanoma-differentiation-associated protein 5 (MDA5), and TLR family members (TLR7 and TLR8) have been characterized as PRRs of HIV-1 RNAs [27,35]. TLR7 was the first reported PRR that detects HIV-1 ssRNA present in the endocytic compartment in plasmacytoid dendric cells (pDCs) as a result of abortive infection due to endocytosis of HIV-1 [33,38]. Further studies demonstrated that TLR7 preferentially recognizes the guanosine (G)- and uridine (U)-rich ssRNA oligonucleotides to initiate production of pro-inflammatory cytokines and chemokines [34].

RIG-I and MDA5, both localized within the cytoplasm, are also reported to activate IFN signaling by detection of both dimeric and monomeric forms of HIV-1 RNA in infected macrophages [23,44]. Stimulation of monocyte-derived macrophages with purified genomic HIV-1 RNA or synthesized HIV-1 ssRNA-derived oligos results in RIG-I-dependent signaling activation. However, in the infected cells, HIV-1 genomic RNA which contains a 5′ cap structure and a polyadenylated (poly-A) tail, resembling cellular RNAs, is a poor PAMP for RIG-I, which has a high affinity for 5′ ppp ssRNA [44]. Moreover, HIV-1 RNAs are enclosed within a viral capsid core, and thus are likely shielded from the innate immune sensors. Uncoating of the capsid occurs only upon initiation of reverse transcription, resulting in possibly no or minimal viral RNA exposure to RIG-I [23,24]. Therefore, RIG-I mediated signaling may play a limited role in triggering antiviral immunity during early steps of viral infection.

Once integrated, numerous transcripts are generated from proviruses by hijacking the cellular transcription machinery. Several members of the host DEAD box helicase (DDX) family such as DDX1, DDX3X, and DDX5 have been characterized as supportive factors to facilitate nuclear export of HIV-1 transcripts, and de novo synthesis of viral protein via binding with different affinities to HIV-1 transcripts [45,46,47]. Intriguingly, in addition to mature viral mRNAs that possess an m^7^GTP cap structure and poly(A) tails, DDX3X was recently found to directly bind to prematurely terminated transcripts, including *tat* transcripts lacking their 3′ poly(A) tail, to trigger an antiviral immune response in monocyte-derived dendritic cells (MDDCs) [29]. Upon binding to abortive HIV-1 RNA transcripts, DDX3X is redistributed to mitochondria and associates with the mitochondrial antiviral-signaling protein (MAVS) to induce IFN responses, while DDX3X associated with mature HIV-1 mRNAs remains assembled in translation units without redistribution. It remains to be determined how binding to different RNA species influences DDX3X subcellular localization. Conversely, HIV-1 actively interferes with the signaling downstream of DDX3X via induction of dendritic cell-specific intercellular adhesion molecule-3-grabbing non-integrin (DC-SIGN) signaling. During HIV-1 infection, DC-SIGN signaling becomes activated via binding of the HIV-1 envelope protein gp120 to DC-SIGN. Subsequently, HIV-1-induced DC-SIGN signaling activates the mitotic kinase PLK1 (Polo-like kinase 1), which is able to impede TRAF3 recruitment to MAVS, leading to attenuation of antiviral immune responses [29]. It is also well documented that activation of DC-SIGN signaling is beneficial to viral transcription elongation [48]. Thus, DC-SIGN signaling is hijacked by HIV-1 to not only boost its transcription, but also evade DDX3X-mediated antiviral immune responses. More recently, two elegant studies showed that intron-containing RNA transcribed from the HIV-1 provirus activates innate immune signaling in MDDCs, macrophages, and CD4^+^ T cells in response to HIV-1 infection. Interestingly, both RIG-I and MDA5, viral sensors that detect cytosolic viral RNAs are not required, whereas the key RLR-adaptor MAVS is essential for signaling transduction [36,37]. Since DDX3X serves as a signaling scaffold to trigger the MAVS-dependent signaling cascade, as described above, it remains to be determined if DDX3X or another uncharacterized RNA sensor upstream of MAVS is responsible for sensing HIV-1 intron-containing RNA for activation of the innate immune response.

### 2.2. Innate Immune Sensors of HIV-1 Reverse Transcription Products

HIV-1 reverse transcription intermediates such as cDNA, ssDNA, DNA/RNA hybrids, and dsDNA are generated and potentially sensed by cytoplasmic DNA sensors, which include cGAS, DDX41, and interferon gamma inducible protein 16 (IFI16) [16,22,40,43,49,50]. IFI16, a member of the PYHIN family, was among the first DNA sensors to be discovered to sense HIV-1-derived DNA products in macrophages and tonsillar CD4^+^ T cells [22,43]. IFI16 preferentially detects incomplete HIV-1 DNA reverse transcripts that accumulate in the cytoplasm of abortively infected tonsillar lymphoid cells to induce inflammasome-mediated cytokine responses [43]. Interestingly, it was reported that upon binding to HIV-1 cDNA, IFI16 recruits stimulator of interferon genes (STING) to activate the TANK-binding kinase 1 (TBK1)– interferon regulatory factor 3 (IRF3) signaling axis, leading to the subsequent transcription of antiviral genes in myeloid cells, whereas, IFI16 activates the inflammasome pathway through ASC and caspase-1, leading to IL-1β production and increased CD4^+^ T cell death in tonsillar lymphoid cells [22,43]. More recently, IFI16 was also found to suppress HIV-1 infection via interfering with the host transcription factor Sp1-dependent viral gene expression in an IFN signaling independent manner [51]. Taken together, these studies suggest that IFI16 has cell-type dependent, as well as multifactorial, roles, first as a DNA sensor that activates innate immune response and second, as a direct antiviral factor that suppresses HIV-1 infection [22,43,51].

During reverse transcription of HIV-1 genomic RNA into cDNA, an RNA/DNA hybrid is created followed by cDNA formation. DDX41, an RNA helicase protein thought to function in RNA splicing, was recently identified as the sensor that primarily binds the short-lived murine leukemia virus (MLV) RNA/DNA hybrids, intermediate products of the reverse transcription [40]. Binding with RNA/DNA hybrids leads to activation of downstream signal transduction in primary mouse macrophages and DCs in a STING-dependent manner [40]. However, it remains to be determined how DDX41 activates STING-mediated signaling, especially in the context of HIV-1 infection. Notably, cGAS upon DNA binding catalyzes the formation of the second messenger cyclic GMP-AMP (cGAMP), which binds and activates STING for activation [16,52], while DDX41 lacks such enzymatic activity to produce cGAMP. Thus, this promised to be an interesting area for future investigation.

Among all the identified DNA sensors detecting HIV-1 infection, cGAS is widely recognized as the major sensor to mount antiviral responses in HIV-1 infected cells [16]. Evidence has shown that cGAS preferentially recognizes the stem-loop structures of single-stranded DNA (ssDNA) derived from HIV-1 cDNA in a sequence-dependent manner, leading to antiviral responses in macrophages [53]. Further studies demonstrated that reverse transcription is required for the IFN response via activation of cGAS [15,16,42]. Blockade of reverse transcription with Nevirapine (NVP), an RT inhibitor, suppresses IFN production in HIV-1 infected MDDCs [15,16,42,54]. Interestingly, integration was also reported to play a role in the IFN response induced by HIV-1 infection as evidenced by the attenuated IFN induction in HIV-infected MDDCs upon treatment with Raltegravir (Ral), an integrase inhibitor [42]. It is still unclear what the exact ligand of cGAS is that triggers the activation of IFN signaling upon after HIV-1 integration. The DNA damage induced by HIV-1 integration might be linked to cGAS-mediated signaling activation. Recently, it has been reported that DNA damage can induce nuclear translocation of cGAS, which is subsequently recruited to the double-stranded breaks to suppress homologous recombination [54]. Therefore, nuclear cGAS might be responsible for innate immune sensing of HIV-1 integration by recognizing self-DNA from damaged chromatin [55,56].

## 3. Regulation of cGAS-STING Signaling in Response to HIV-1 Infection

### 3.1. Co-Factors of HIV-1-Mediated cGAS-STING Pathway Activation

**Figure 1:**
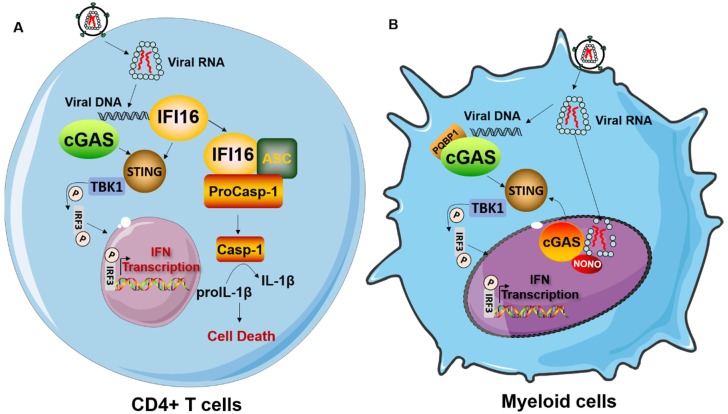
Innate immune sensing of HIV-1 in CD4^+^ T cells and myeloid cells. (**A**) HIV-1 infection in CD4^+^ T cells leads to induction of IFNs and the inflammatory response. cGAS senses incomplete reverse transcription (RT) products to elicit the IFN response via the STING-TBK1-IRF3 axis. While IFI16 recognizes abortive RT products to induce IFNs as well as inflammasome activation by binding to the adapter molecule ASC (apoptosis-associated speck-like protein containing a CARD). This leads to activation of caspase-1 and the cytokine IL-1β, triggering pyroptosis. (**B**) In myeloid cells, cGAS acts as the main sensor to detect HIV-1 PAMPs. cGAS signals through STING to drive downstream signaling activation. polyglutamine binding protein-1 (PQBP1) serves as a proximal sensor of the cGAS-dependent innate response to HIV-1 infection within the cytoplasm, while non-POU (Pit-Oct-Unc) domain-containing octamer-binding protein (NONO) promotes cGAS-mediated innate immune activation through binding of the HIV-1 capsid protein and cGAS within the nucleus.

cGAS-STING signaling has been considered a major sensing pathway to mount the antiviral immune response in the context of HIV-1 infection. Therefore, in this section, we will elaborate on the mechanisms regulating cGAS-STING signaling activation in response to HIV-1 infection. Since its discovery, numerous findings regarding the regulation of cGAS-STING signaling activation have been described [57,58,59,60] and summarized [61]. For instance, post-translational modifications of cGAS such as phosphorylation, SUMOylation, ubiquitination, and glutamylation have been documented to regulate cGAS/STING signaling activation [57,58,59,60]. However, these cGAS regulating mechanisms have been poorly characterized in the context of HIV-1 infection. Among all of the well characterized cGAS regulators, polyglutamine binding protein-1 (PQBP1) and Non-POU (Pit-Oct-Unc) domain-containing octamer-binding protein (NONO) are the only host factors currently known to specifically regulate cGAS-mediated innate sensing of HIV-1 infection (Figure 1) [15,21,62,63]. PQBP1 is a highly conserved protein which is mutated in Renpenning syndrome, an X-linked mental retardation disorder [64]. It was identified as a proximal sensor of cGAS-mediated signaling in response to HIV-1 infection through a targeted RNAi screen in MDDCs [15]. Biochemical studies demonstrated that PQBP1 binds to cGAS as well as HIV-1 reverse-transcribed DNA to enhance the IFN response in HIV-1 infected MDDCs. The WW domain within the N-terminus of PQBP1 has been shown to be important for cGAS interaction, while the C-terminal domain of PQBP1 is necessary for its association with HIV-1 cDNA and to induce cGAS signaling. It appears that PQBP1 bridges HIV-1 DNA recognition and cGAS activation [15]. Interestingly, knockdown of PQBP1 only impairs innate signaling in response to infection by HIV-1 and other retroviruses, but has no effect on the signaling induced upon infection with DNA viruses or transfection of DNA [15]. Therefore, PQBP1 exhibits specificity for regulating retrovirus-mediated cGAS-STING signaling activation.

NONO, another proximal sensor of the cGAS-dependent HIV-1 innate response in MDDCs was recently characterized [21]. In contrast to PQBP1, which functions in the cytosol to promote the cGAS-mediated sensing of HIV-1 within infected MDDCs, NONO enhances cGAS-dependent HIV-1 DNA sensing in the nucleus. Deletion of NONO greatly impairs the abundance of cGAS in the nucleus, but it has no impact on the cytosolic pool of cGAS. More importantly, NONO directly binds to the HIV-1 nuclear monomeric capsid and cGAS to trigger signaling activation. A conserved region in the HIV-1 capsid with limited tolerance for escape mutations was mapped as the crucial motif required for binding NONO [21].

There may be several reasons as to why innate recognition of HIV-1 and other retroviruses require adaptor proteins that are dispensible for immune response to DNA viruses. cGAS responds to threshold levels of accumulated pathogen-associated DNA, thus avoiding abberant activation to self-DNA specifies (i.e., leakage of mitochondrial or nuclear DNA). Unlike DNA viruses, whose DNA genomes are amplified upon infection, unintegrated HIV DNA is of limited abundance and ephemeral. Thus, mounting a robust and specific response to transient and low abundance retrovirally-encoded DNA represents a major challenge for the innate immune system. These adaptor proteins may enable cGAS to recognize and authenticate transient and low abundance DNA PAMPs that are unique to retroviral infection.

### 3.2. Negative Regulators of HIV-1-Mediated cGAS-STING Pathway Activation

In addition to these positive regulators required for cGAS-STING signaling activation in response to HIV-1 infection, several host factors were reported to negatively regulate cGAS-mediated signaling in the context of HIV-1 infection [61,65]. The nucleotide oligomerization domain (NOD)-like receptor X1 (NLRX1) was previously identified in a global siRNA screen as an HIV-1 dependency factor, although with an unknown function [66]. Further studies demonstrated that NLRX1 suppresses the IFN response by preventing STING and TBK1 interaction in the mitochondrial-associated membrane, thereby negatively regulating STING-dependent signaling and the innate response to HIV-1 and other DNA viruses [67]. Coincident with these in vitro findings, studies performed in macaques infected intravaginally with SIV showed that NLRX1 is upregulated very early upon inoculation and inversely correlated with expression of antiviral ISGs expression, further underscoring the importance of NLRX1 in HIV-1 infection and transmission by manipulating the antiviral immune system [67,68]. More recently, NLRC3 (NLR Family CARD Domain Containing 3), another member of the NOD-like receptors family was found to be a negative regulator that attenuates the IFN response by sequestering and attenuating STING activation, similar to NLRX1 [69]. Upon stimulation, NLRC3 directly binds viral DNA and other nucleic acids through its leucine-rich repeat (LRR) domain in a sequence-independent manner. DNA binding to NLRC3 increases its ATPase activity, and causes the dissociation of NLRC3 and STING, thus licensing an IFN-I response [69]. Consequently, NLRC3 might be involved in modulating cGAS/STING signaling during HIV-1 infection, although it is currently not known if HIV DNA binds to NLRC3 and promotes its dissociation from STING.

### 3.3. Innate Control of Cytoplasmic DNA Accumulation

Accumulation of excess cytoplasmic DNA is a prerequisite for cGAS activation to elicit IFN production [70]. Host factors such as SAMHD1 (SAM domain and HD domain-containing protein 1), and TREX1 (Three Prime Repair Exonuclease 1), are the key players in limiting the accumulation of cytoplasmic DNA that can be sensed by cGAS [70,71]. Initially, SAMHD1 was discovered as a restriction factor that prevents HIV-1 reverse transcription through depletion of cellular dNTPs in myeloid cells [18,19]. More recently, it was also found that SAMHD1 is a key regulator in innate immune sensing [72,73,74]. SAMHD1 can prevent the cytosolic accumulation of ssDNA by promoting the degradation of nascent DNA at stalled replication forks, which may escape the nucleus during mitosis, thereby limiting innate immune sensing by cGAS [73]. Another study reported that SAMHD1 is able to directly interact with the inhibitor-κB kinase ε (IKKε) and IRF7 to suppress the innate immune response by reducing IKKε-mediated IRF7 phosphorylation [72,74]. Thus, the presence of SAMHD1 in myeloid cells infected with HIV-1 might be beneficial for viral replication in MDDCs and transmission to CD4^+^ T cells by suppressing the HIV-1-induced innate immune response [75,76,77].

The ubiquitously expressed host exonuclease TREX1 is also crucial for preventing activation of the cGAS/STING/IRF3 signaling axis by eliminating cytoplasmic DNAs before their detection by cGAS [49,71]. A recent report showed that the intracellular level of TREX1 is negatively correlated with the production of IFNs and ISGs during the early events of HIV-1 infection, thus, underscoring the pivotal role of TREX1 in the HIV-1 mediated innate response [28]. As a result of TREX1 and SAMHD1 depletion, HIV-1 cDNA is accumulated and triggers the cGAS-dependent IFN response. Intriguingly, observations in elite controllers (EC), a patient group controlling HIV-1 in the absence of treatment, point to a protective role of weak induction of SAMHD1 [8]. Primary conventional dendritic cells (cDCs) of elite controllers mount a much more effective innate response and display greater CD8^+^ T-cell control of infection in comparison to cDCs from other patient groups. This response is accompanied by reduced induction of SAMHD1 specifically in cDC from ECs, whereas TREX1 levels were significantly upregulated upon HIV exposure in cDCs from all patient groups except highly active antiretroviral therapy (HAART)-treated patients [8]. Therefore, the balance between the HIV-1 infection rate and protein abundance of these exonucleases might determine the magnitude and duration of antiviral innate immunity in HIV-1 infected cells.

### 3.4. HIV-1 Capsid Is a Viral Determinant of Innate Sensing of HIV-1

In addition to these host factors involved in innate immune sensing of HIV-1 infection, the HIV-1 capsid, a protein shell composed of hexamer and pentamer structural units of viral CA protein, has emerged as a determinant of innate sensing of HIV-1 infection [14,21,78,79]. Specifically, it has been reported that the interactions between the HIV-1 capsid with cleavage and polyadenylation specificity factor subunit 6 (CPSF6) or host cyclophilin A (CypA) influence the activation of the innate immune response [20,80,81]. HIV-1 CA mutant N74D, which is incapable of binding to CPSF6, elicits a stronger innate activation in macrophages, compared to wild-type HIV-1 [20]. An elevated IFN response was also observed in MDMs infected with the CypA binding-deficient CA mutant (P90A). Although the mechanism of action is not fully defined yet, it appears that the HIV-1 capsid prevents the innate sensing of HIV-1 infection by hijacking multiple host factors [20,82]. Based on reports that the association with CPSF6 or CypA enhances the rigidity and stability of the HIV-1 capsid, a capsid cloaking model, whereby HIV-1 capsid shrouds the viral genomic material from pattern recognition, has been proposed [14,20].

However, other studies surprisingly found that the HIV-1 capsid also may act as a PAMP that is recognized by multiple host factors including tripartite motif-containing protein 5 (TRIM5) and NONO [21,79]. TRIM5, a RING domain-E3 ubiquitin ligase, was reported to specifically interact with the HIV-1 capsid lattice. The interaction likely enhances BC13/UEV1A-dependent E3 activity of TRIM5, which leads to the formation of unattached ubiquitin chains that can directly activate protein kinase enzymes such as TAK1. As a consequence, activated TAK1 mediates innate immune signaling transduction by activating NF-κB and activator protein 1 (AP-1). As described above, NONO is also reported to recognize dissociated HIV-1 capsid in the nucleus to promote the HIV-1-induced IFN response. Interestingly, NONO was also found to bind to HIV-1 capsid protein with lower affinity than the capsid from the weakly pathogenic HIV-2 [21]. The results potentially explain why HIV-2, rather than HIV-1, is efficiently sensed in DCs to trigger the innate immune response before integration [14]. These data also challenged the capsid cloaking model proposed previously [14,20], but the relative contribution of HIV-1 capsid as a determinant of HIV-1 innate sensing will require further investigations.

## 4. The Cell Type-Dependencies of Innate Immune Response to HIV-1 Infection

### 4.1. Innate Immune Response to HIV-1 Infection in DCs and Macrophages

Innate immune sensing of HIV-1 infection is also determined by the target cell types generated during HIV-1 infection (Figure 1). As described above, the differential expression of host factors involved in innate sensing in different cell types contributes to the magnitude and duration of ensuing signaling. Macrophages and DCs belong to the mononuclear phagocyte system (MPS), which performs an important immune surveillance function against intracellular pathogens [83]. Compared to activated CD4^+^ T cells, both macrophages and DCs are less susceptible to HIV-1 infection, largely due to high expression of the restriction factor SAMHD1 [18,19]. Despite poor replication in macrophages and DCs, induction of a detectable IFN response after HIV-1 infection has been observed [15,16,39,84]. In MDDCs and macrophages, HIV-1 infection induces a robust IFN response in a manner dependent on the cGAS-STING pathway [16,42]. As discussed above, the addition of viral-like particles (VLPs) carrying Vpx enhances HIV-1 infection and promotes cGAS-dependent innate immune response through degradation of SAMHD1 and possibly other undefined mechanisms [14,15]. In pDCs, HIV-1 primarily triggers an RNA induced antiviral response through TLR7 activation [39,84,85]. However this response is dependent on endocytosis, and thus is not a byproduct of productive infection. The specific relevance of cGAS-STING signaling in sensing of HIV-1 infection in infected pDCs is still unclear, although human pDCs harbor a functional cGAS-STING pathway [86].

### 4.2. Innate Immune Response to HIV-1 Infection in CD4^+^ T Cells

CD4^+^ T cells normally coordinate the adaptive T- and B-cell response to defend against invading pathogens. The replication life cycle of HIV-1 within CD4^+^ T cells is well studied in molecular detail. However, to what extent innate sensing occurs in CD4^+^ T cells during HIV-1 infection is still under debate. Recently, it was shown that the cytosolic DNA sensor cGAS is also implicated in sensing HIV-1 infection in activated CD4^+^ T cells [41]. Further characterization has indicated that productive infection, particularly proviral DNA integration, is required for cGAS-dependent DNA sensing in activated CD4^+^ T cells [41,87]. Additionally, IFI16 has been reported to act as a specific sensor, detecting abortive HIV-1 reverse transcripts to invoke caspase-1 activation and pyroptosis in tonsillar lymphoid CD4^+^ T cells abortively infected with HIV, as previously described [43]. Thus, these studies support the concept that innate sensing of HIV-1 infection occurs in CD4^+^ T cells, the principal targets of HIV-1. However, other studies have shown that innate sensing and the IFN response were virtually absent in HIV-1 infected CD4^+^ T cells in vivo [88,89]. Moreover, during HIV-1 infection of primary CD4^+^ T cells, it has been reported that either cGAS is not essential for IFN induction [90], or significant IFN levels are not even induced [91]. The different experimental settings and/or status of isolated CD4^+^ T cells may account for these contradictory results, and additional investigation of innate immune surveillance of HIV-1 in CD4^+^ T cell will be necessary to understand retroviral sensing in the lymphoid compartment.

## 5. Strategies Adopted by HIV-1 to Avoid Sensing and IFN Response

### 5.1. Counteraction of HIV-1 Restriction Factors

Although humans are equipped with a variety of intrinsic, innate, and adaptive antiviral immune responses, HIV-1 has evolved sophisticated evasion strategies to specifically counteract the cellular antiviral defense [92,93]. Once activated, sensing pathways ultimately result in increased expression of host restriction factors. A primary mechanism by which lentiviruses, including HIV-1, evade innate immune restriction carried out by antiviral factors is through hijacking of cellular ubiquitin ligases to ubiquitinate host restriction factors and target them for proteasome-mediated degradation [93,94,95]. HIV encoded accessory proteins are the predominant mediators of this immune evasion strategy. HIV-1 Vif recruits the Cul5/Elongin ubiquitin ligase complex to target APOBEC3 for degradation [96,97,98], while Vpu downmodulates both CD4 and tetherin/BST2 by recruiting a cullin1-Skp1 ubiquitin ligase complex. Recently, it was found that HIV-1 Vpu targets additional ISGs including CD99, PLP2, and UBE2L6 for proteolytic degradation in a manner mechanistically similar to antagonism of BST2 [99,100,101,102]. HIV-1 Vpr and its Vpr/Vpx homologs in HIV-2 and SIV associate with the cullin4A-DDB1-DCAF1 complex to target multiple host factors including tet methylcytosine dioxygenase 2 (TET2) and helicase-like transcription factor (HLTF) for degradation, thus promoting virus infection [103,104]. This interaction is also essential for Vpx-induced degradation of SAMHD1, facilitating reverse transcription in myeloid and resting T cells, as mentioned above [18,19,105]. In addition to CD4 and major histocompatibility complex class I (MHC-I), HIV-1 Nef recently was found to counteract host transmembrane proteins SERINC5 and SERINC3 through excluding these restriction factors from virion incorporation [106,107]. However, tailoring a customized response to a given antiviral protein is not a very efficient process, given the plethora of antiviral factors and the limited toolset encoded by the small HIV-1 genome. A number of strategies for HIV-1 mediated evasion of innate immune mechanisms through blockade of sensing and signaling pathway transduction are beginning to emerge.

### 5.2. Disruption of Signaling Pathway Transduction

Another stage where HIV-1 can interfere with the innate immune response is the signaling event itself that follows recognition of a PAMP. There is increasing evidence that HIV-1 causes global changes in the host transcriptional network to manipulate cellular responses including the innate immune response [108]. Although it is well established that HIV-1 uses its Vpu protein to counteract NF-κB signaling, the impact on the global transcriptome, especially genes related to immune responses, remained unclear [109,110]. Recently, global transcriptional profiling of primary CD4^+^ T cells, infected with three different primary HIV-1 isolates, showed that compared to their wild type counterpart, *vpu*-deficient viruses lost their capability to suppress NF-κB target genes and triggered a much stronger IFN response. Langer et al. further showed that Vpu-mediated counteraction of NF-κB signaling suppressed the expression of restriction factors and release of IFNs [26]. Conflicting data exists on the role of Vpu in regulating IRF3 [51,111]. In one study, HIV-1 Vpu protein was demonstrated to attenuate innate antiviral immunity in HIV-1-infected cells through targeting IRF3 proteolysis in lysosomes. HIV-1 Vpu was shown to bind to cytoplasmic IRF3 and direct its translocation into endolysosomal compartment, thus facilitating IRF3 degradation [112]. In contrast, Harman et al. found that HIV-1 does not antagonize IRF3-dependent signaling by targeting IRF3 for degradation, instead, HIV-1 blocks TBK1 phosphorylation via two different viral accessory proteins Vpr and Vif in DCs and macrophages [113]. However, Vpr seems to display the opposite effect, and potentiates the type I IFN response in HIV-1 infected CD4^+^ T cells, while the other accessory protein Vpu efficiently suppressed the IFN response in infected CD4^+^ T cells through an undefined mechanism [41]. Therefore, multifaceted strategies are likely required to block the immediate antiviral response to ensure establishment of systemic infection and evading adaptive responses.

### 5.3. HIV-1 PAMPs Masking

Cloistering its PAMPs represents an efficient way for HIV-1 to prevent sensing, but cells have evolved elegant strategies to uncover PAMPs. The cell uses a post-transcriptional modification called 2′-*O*-methylation, catalyzed by 2′-*O*-methyltransferases (2′*O*-Mtases) as a means to distinguish foreign RNA from its own “self” RNA. The cellular RNAs, including mRNAs, ribosomal RNAs, small nuclear RNAs, and transfer RNAs are all subjected to 2′-*O*-methylation, whereas exogenous RNAs, such as viral RNA, are missing this feature and can thus be identified to then trigger a type I IFN response [114]. To escape the innate immune recognition by the host, some viruses, such as Zika virus and Ebola virus express their own 2′*O*-Mtase to modify their RNA. While HIV-1 does not express such an enzyme, a recent study showed that HIV-1 still overcomes this obstacle by recruiting the cellular 2′*O*-Mtase in a complex with TAR RNA-binding protein (TRBP) to the viral RNA, thereby acquiring 2′-*O*-methylation at multiple distinct sites [27]. HIV-1 particles produced in HEK293T cells depleted of FTSJ3, cellular a 2′*O*-Mtase, showed reduced or even complete abrogation of 2′-*O*-methylation compared to wild type HEK293T cells and triggered a drastically increased release of IFN-α and IFN-β in transfected or infected pro-monocytic U937 cells. Interestingly, the RNA sensor MDA5, but not RIG-I, appeared to be necessary for detecting the unmethylated HIV-1 RNA and triggering innate immune activation [27].

Another way to reduce the risk of being detected by innate sensors is to minimize the time HIV-1 lingers in the cytoplasm, exposing itself to innate sensors. To migrate through the dense cytoplasm of an infected cell, HIV-1, as well as other viruses, are known to take advantage of the microtubule motor protein dynein for retrograde trafficking towards the nucleus [115]. Recently, Dharan et al. showed that HIV-1 uses its capsid to bind the dynein adaptor protein bicaudal D2 (BICD2) to engage with dynein and co-opt the microtubule system. Depletion of *BICD2* in TZM-bl cells perturbed the cytoplasmic trafficking of HIV-1 during infection and left HIV-1 more vulnerable to detection by innate immune sensors [116]. These results emphasize the necessity for HIV-1 to get to the nucleus as quickly as possible to avoid sensing in cytosol.

## 6. Summary

The innate immune system furnishes a front-line defense against pathogenic infection through its direct antiviral action or its indirect induction of primary adaptive immune responses. Our knowledge of the molecular mechanisms and pathways of innate immune activation during HIV-1 infection has improved significantly in recent years. However, many important details of the processes and consequences of innate immune activation in HIV-1 target cells remains poorly defined (Box 1). There is still no clear knowledge of the structure, molecular features, and accessibility of the HIV-1 PAMP, as well as the definitive innate sensing pathway(s) and cell types that shape the cellular and humoral immune responses during HIV-1 infection, and its impact in HIV-1 transmission. The available evidence suggests that innate sensing primarily occurs in DCs and macrophages upon infection through detection of cytoplasmic DNA and endosomal RNA. Yet, how the activation of innate immune responses in DCs and macrophages influences the likelihood of CD4^+^ T cell infection and subsequent spread of virus remains elusive. Similarly, it remains to be understood how activation of these professional antigen presenting cells (APCs) shape HIV-specific humoral responses, including CD4^+^ and CD8^+^ T cell activation. Previous studies have revealed that the innate sensing cGAS/STING pathway in APCs functions to augment the spontaneous generation of activated CD8^+^ T cells [117,118,119]. Thus, harnessing innate immune activation by targeting the innate sensors in APCs with innate immunostimulatory agents may have substantial therapeutic benefits. Moreover, since sexual transmission is the dominant mode of HIV-1 acquisition, and mucosal DCs and macrophages are the primary sentinels that are able to capture and sense exposed virions, understanding the innate sensing and activation in the mucosa might be a critical component for devising novel strategies to prevent infection. Finally, the role of the innate immune response in additional aspects of HIV-1 pathology remains an open question. For example, it is unclear what role the innate immune system plays in the seeding of the viral reservoir, as well as potential contributions to latency reactivation and viral clearance. Taken together, it will be necessary to continue to investigate in more detail the regulatory circuits that govern innate immune sensing of HIV-1 in multiple disease relevant cell types. Unraveling these molecular mechanisms will illuminate the molecular basis for the cellular control of viral transmission and systemic infection, as well as inform the design of next generation vaccines and adjuvants that will enable the eradication of HIV-1 infections across the globe.

Box 1 **Key questions or challenges in HIV-1 innate sensing field.**

- Understanding the role of the innate immune response in the control of viral transmission, systemic infection, and potentially latency.
- Defining the structure, molecular features, and accessibility of the HIV-1 DNA PAMP.
- Dissecting the distinct roles of PQBP1, NONO, and other cellular co-factors in cGAS signaling activation in response to HIV-1 infection.
- Gaining a thorough mechanistic understanding of innate sensing of HIV-1 infection in CD4+ T cells.
- Mechanisms of HIV-1-mediated evasion of innate sensing.
- Harnessing knowledge of the innate response to develop novel vaccines, vaccine adjuvants, as well as prophylactic and therapeutic approaches to prevention of HIV infection.

## Figures and Tables

**Table 1:** Pathogen-associated molecular patterns (PAMPs) and sensors involved in innate sensing of human immunodeficiency virus type 1 (HIV-1) infection.

Infection Course	PAMP	Sensor	Signal Axis	Immune Effector Response	Cell Type	Reference
Entry, Uncoating	Single-stranded RNA (ssRNA)	RIG-I/MDA5	MAVS-TRAF3-TBK-IRFs	Inflammatory cytokines Type I IFNs	Macrophages	[23,27,35]
	Single-stranded RNA (ssRNA)	TLR7/TLR8	MyD88-TRAF6-NF-κB	Inflammatory cytokines Type I IFNs	Plasmacytoid DCs	[24,32]
	Viral Fusion	Unknown	Unknown	Type I IFNs	MDMs	[39]
Uncoating, Reverse Transcription	RNA/DNA hybrids	DDX41	STING-TBK1-IRFs	IFNs	BMDMs BMDCs	[40]
	Double-stranded DNA (dsDNA)	cGAS	STING-TBK1-IRFs	Inflammatory cytokines and IFNs	DCs Macrophages CD4^+^ T cells	[16,41,42]
	Double-stranded DNA (dsDNA)	IFI16	ASC-proCasp-1-IL1β	Caspase-1 activation Pyroptosis	Quiescent CD4^+^ T cells	[43]
	Single-stranded DNA (ssDNA)	IFI16	STING-TBK1-IRFs	IFNs	Macrophages Activated CD4^+^ T cells	[22]
Integration	Unknown	cGAS	STING-TBK1-IRFs	IFNs	MDDCs CD4^+^ T cells	[41,42]
Transcription Translation	Intron-containing RNA	Unknown	MAVS-TRAF3-TBK-IRFs	IFNs	MDMs CD4^+^ T cells	[36,37]
	Abortive viral RNA	DDX3X	MAVS-TRAF3-TBK-IRFs	IFNs	DCs	[29]
